# Central amygdala *Fkbp5* expression correlates with faster submission and ethanol self-administration reacquisition: Benztropine reduces ethanol relapse-like reacquisition in stressed rats

**DOI:** 10.1016/j.ynstr.2025.100770

**Published:** 2025-11-08

**Authors:** Luisa B. Bertotto, Eleanna M. Sakoulas, Marian L. Logrip, Katrina Lin, Anastasia E. Pimentel, Lenwood Thompson, Bryan Cruz, Valentina Vozella, Cristiane A. Favoretto, Marisa Roberto, Eric P. Zorrilla

**Affiliations:** a Department of Translational Medicine, The Scripps Research Institute, La Jolla, CA, 92093, USA; b Division of Biological Sciences, University of California, San Diego, 92093, USA; c Department of Psychology, Indiana University Indianapolis, Indianapolis, IN, 46202, USA; d Indiana Alcohol Research Center, Indiana University School of Medicine, Indianapolis, IN, 46202, USA; e Division of Cognitive Sciences and Psychology, University of California, San Diego, 92093, USA

**Keywords:** Ethanol or alcohol use disorder, Social defeat or foot-shock stress, FK506-Binding protein 51 or FKBP51, Relapse, Benztropine or cogentin, Corticosteroid receptor, Central nucleus of the amygdala, *Fos*, *FosB*, *ΔFosb* immediate-early genes, Sex difference

## Abstract

Stress-related emotional disorders, such as post-traumatic stress disorder (PTSD) and major depression, increase alcohol relapse risk. PTSD, depression, and alcohol use phenotypes associate with gene variants of FKBP prolyl isomerase 5 (*FKBP5*), a chaperone modulator of glucocorticoid receptors (GR). FKBP51 inhibitors can decrease ethanol intake, but FKBP51's role in recurrence of post-stress ethanol drinking is unknown. We tested the hypotheses that expression of *Fkbp5* and immediate early genes (IEGs) in the central nucleus of the amygdala (CeA) is increased in rats with a history of defeat or foot-shock stress and associates with faster submission and increased reacquisition of ethanol self-administration. We tested if benztropine mesylate, an FDA-approved drug that inhibits FKBP51-GR binding, reduces reacquisition of ethanol self-administration in rats with a history of foot-shock stress. Wistar rats were studied after resident-intruder social defeat (*n* = 32) or in an ethanol self-administration reacquisition model, with or without repeated foot-shock history (*n* = 62). Acute social defeat stress increased CeA IEG expression within 1 h *Fkbp5* expression by 6 h. CeA IEG activation correlated with *Fkbp5* expression, and both correlated with faster submission to defeat. CeA *Fkbp5* expression also associated with greater ethanol intake and blood ethanol concentration during reacquisition of ethanol self-administration. Benztropine (i.p., 5, 10 mg/kg) dose-dependently reduced relapse-like ethanol reacquisition, and sex-specific analyses suggest a more robust effect in males than females. The results warrant the study of CeA FKBP51 in passive stress coping and of drug-like selective FKBP51 inhibitors to reduce ethanol relapse after histories of repeated stress.

## 1 Introduction

Alcohol use disorder (AUD) is a disabling, deadly, and costly disease (Grant et al., 2017) with high recidivism. AUD relapse rate is heightened in individuals with post-traumatic stress (Driessen et al., 2008; McCarthy and Petrakis, 2010; Norman SB et al., 2007) and depressive disorders (Curran et al., 2000; Hasin et al., 2002; Oliva et al., 2018). Post-traumatic stress disorder (PTSD) and depression also are each associated with increased alcohol use and comorbid AUD (Blanco et al., 2013; Brière et al., 2014; Debell et al., 2014; Shorter et al., 2017). Past stress is an etiologic factor in both disorders, and, in animal models, past chronic stress also increases vulnerability to stress-induced alcohol relapse-like behavior (Becker et al., 2023). Our laboratory similarly found that after extinction from ethanol (EtOH) self-administration, rats with a history of repeated foot-shock stress showed increased reacquisition (Logrip et al., 2014; Logrip and Zorrilla, 2012). Treatments that reduce alcohol relapse after a history of stress are needed.

FK506-binding protein 51 (FKBP51; International Union of Basic and Clinical Pharmacology [IUPHAR] nomenclature), a glucocorticoid receptor (GR) co-chaperone, is a promising target to counteract overactivity in molecular substrates shared by stress and alcohol use (Dragan et al., 2018; Kang et al., 2019; Xie et al., 2010; Zannas et al., 2016). FKBP51 has been hypothesized to play a key role in (mal)adaptive responses to stress. Increased FKBP51 activity is associated with reduced negative feedback inhibition of the stress axis (Binder, 2009), increased circulating corticosterone, and reduced stress-induced GR translocation and transcriptional adaptation. After adverse life events, variants for *FKBP5,* the human gene that encodes FKBP51 (Human Gene Organization [HUGO] nomenclature), influence risk for subsequent PTSD (Binder et al., 2008; Boscarino et al., 2012; Hawn et al., 2019; Klengel et al., 2013; Li et al., 2019; Watkins et al., 2016; Xie et al., 2010; Zhang et al., 2020) and depression (Appel et al., 2011; Dackis et al., 2012; Zimmermann et al., 2011). *FKBP5* polymorphisms also associate with problematic drinking (Dragan et al., 2018), alcohol withdrawal severity (Huang et al., 2014), alcohol intake, and AUD symptoms (Qiu et al., 2016a). We recently found that two FKBP51 inhibitors reduced EtOH intake and preference in a rat model of comorbid PTSD/AUD (Cruz et al., 2023). One of the studied FKBP51 inhibitors, benztropine (the FDA-approved drug Cogentin®), which also possesses anti-muscarinic, anti-histaminergic, and dopamine reuptake-inhibiting properties (Rothman et al., 2008), also reduced circulating corticosterone, startle reactivity, and, in females, aggressive irritability (Cruz et al., 2023). Given the reviewed findings, the present study tested the hypothesis that benztropine also could reduce post-extinction reacquisition of EtOH self-administration – an aspect of relapse – in rats with a stress history. Given reported sex differences in FKBP51 function in stress and drinking (Cruz et al., 2023; van Doeselaar et al., 2023), potential sex differences in benztropine action were considered.

We also sought to determine here whether expression of *Fkbp5,* the rat ortholog of *FKBP5* (Rat Genome Database [RGD] nomenclature), in the central nucleus of the amygdala (CeA), which subserves negative emotional responses to stress, withdrawal, and alcohol reinforcement (Blasio et al., 2013; Cottone et al., 2019; Gilpin et al., 2015; Roberto et al., 2017; Serrano et al., 2018; Zorrilla and Koob, 2013, 2019; Zorrilla et al., 2001), correlates with CeA activational responses to stress. In humans, *FKBP5* variants predict both resting-state and stress-induced amygdala activity (Holz et al., 2015; Pagliaccio et al., 2014, 2015a, 2015b). Further, restraint, food deprivation, and glucocorticoids acutely increase CeA *Fkbp5* expression in rodents (Attwood et al., 2011; Scharf et al., 2011). Thus, we tested whether: 1) social defeat increases CeA *Fkbp5* expression, 2) *Fkbp5* expression predicts CeA activation responses to defeat, as defined by the immediate-early genes (IEG) *Fos*, *FosB*, *ΔFosb*, *Egr1*, *Egr2*, *Jun,* or *JunB*. Social defeat was studied as a stressor because, in humans, social defeat, including being bullied, mobbed, or losing social rank, predicts PTSD, anxiety, and depression symptoms (Bjorkqvist, 2001; Siddaway et al., 2015; Tatar and Yuksel, 2019; Troop and Hiskey, 2013). In rodents, resident-intruder models of social defeat elicit both depressive-like symptoms and posttraumatic stress-like symptoms (see Diaz and Lin, 2020; Hammels et al., 2015; Verbitsky et al., 2020 for reviews). The latter include increased fear conditioning (Manz et al., 2018), generalized social fear avoidance (Diaz and Lin, 2020), acoustic startle reactivity (Kinn Rod et al., 2012; Pulliam et al., 2010), neophobia and anxiety-like behavior (Becker et al., 2001; Chen et al., 2024; Harada et al., 2024; Kinn Rod et al., 2012; Meerlo et al., 1996; Skutella et al., 1994), reactivity to mild stressors (Gautam et al., 2018; Koolhaas et al., 1997), sleep fragmentation and awakenings (Ahnaou and Drinkenburg, 2016; Grafe et al., 2020; Kinn et al., 2008; Page et al., 2016), enduring defeat cue-induced muscle tension, hyperarousal, and behavioral inhibition (Nelson et al., 2010; Watt et al., 2009), tachycardia (Koolhaas et al., 1997), post-stress-like gut microbiome changes (Gautam et al., 2018), and peripheral inflammation (Finnell et al., 2017) in subordinated rodents.

Lastly, we studied *Fkbp5* here in relation to passive stress-coping behaviors. “Active” coping behaviors, related to problem-focused coping, have been defined to take direct action to control or resolve the stressor itself (Association, 2018; Brown and Nicassio, 1987; Carroll, 2020). In contrast, “passive” coping behaviors, related to emotion-focused coping, do not address the stressor or problem and instead involve greater self-medication or alcohol use to “cope”, avoidance of the root issue, or acceptance (Broekmans et al., 2010; Cooper et al., 1992; Stoppelbein et al., 2013; Veenstra et al., 2007). *Fkbp5* knockout mice show increased active coping behavior during forced swim (Hoeijmakers et al., 2014; Touma et al., 2011). Thus, we tested the hypothesis that greater CeA *Fkbp5* expression correlated with more passive coping behavior, as defined by faster submission to defeat (Walker et al., 2009; Wood et al., 2013, 2015) or greater reacquisition of EtOH intake (Blanchard et al., 1987; McKenzie-Quirk and Miczek, 2008; Veenstra et al., 2007). Because FKBP51 and GR have been suggested to indirectly regulate expression of one another (Bali et al., 2016; Jääskeläinen et al., 2011; Maiarù et al., 2016), we also studied whether mRNA expression of the signaling glucocorticoid receptor (GR*α*; nuclear receptor subfamily 3 group C member 1, *Nr3c1a*) or its truncated splice variant GR*β*, that has distinct, opposing actions (*Nr3c1b*) (Bamberger et al., 1995; Fruchter et al., 2005; Kino et al., 2009), correlated with defeat submission latency or drinking behavior.

## 2 Methods

### 2.1 Subjects

Adult, individually housed, Wistar rats (Charles River, Raleigh, NC, USA; 7–8 weeks upon arrival) were studied for gene expression after social defeat (*n* = 32 males with no alcohol history), gene expression after foot-shock stress and reacquisition of EtOH self-administration (*n* = 11–12 per stress condition), and relapse-like self-administration after benztropine treatment (n = 21/sex). For social defeat studies, adult, male Long-Evans rats (450–700 g on arrival) that were substantially larger than the Wistar “intruder” rats were used as territorial “residents”, housed in enclosures (48 × 69 × 50 cm) with sawdust-covered, stainless-steel floors. To promote territorial behavior, each resident (*n* = 18) was stably housed for at least 1 month with an adult, female Wistar rat (*n* = 18) that had received electrocauterization of the uterine coils under isoflurane anesthesia (1 %–3 % in oxygen) to prevent pregnancy. Rats were maintained in a temperature- (20–22 °C) and humidity-controlled (30–70 %) vivarium under a reverse light cycle (lights off 8 a.m./on 8 p.m.). Food (LM-485 7012, Teklad Diets-Envigo, Madison, WI, USA) and tap water were available *ad libitum*. All procedures conformed to the National Institutes for Health *Guide for the Care and Use of Animals* and were approved by the Institutional Animal Care and Use Committee of Scripps Research (Protocol 08–0010).

### 2.2 Study 1 - social defeat model

The resident-intruder procedure for experimental social defeat was used (Fekete et al., 2009; Spierling et al., 2017). Adult, male Wistar rats, a strain with moderate social agonistic behavior (Scholtens and Vandepoll, 1987; Schuster, 1993), were “intruders”. Due to the strain's propensity for territoriality and dominance behavior (Becker et al., 2023; Miczek, 1979; Tornatzky and Miczek, 1993), Long-Evans rats were “residents”. To potentiate dominance behavior, residents were exposed to “training” intruders — post-pubertal, smaller male Wistar rats — for 12 days before experimental studies. Training intruders were removed from the home cage immediately post-defeat, and females were returned to the cage. Defeat was defined as adoption of a submissive, supine posture by the intruder/training rat (Miczek, 1979; Tornatzky and Miczek, 1993). Training reduces the mean latency by which residents achieve submissions over experimental intruders to <90 s and reduces the duration of physical conflict that residents require to attain defeats. Potential residents that injured intruders during training, did not achieve 3 consecutive days of defeat, or had mean defeat latencies >120 s were excluded, yielding the 18 residents in the present study. Training was conducted during the dark cycle under red lighting.

**Fig. 1:**
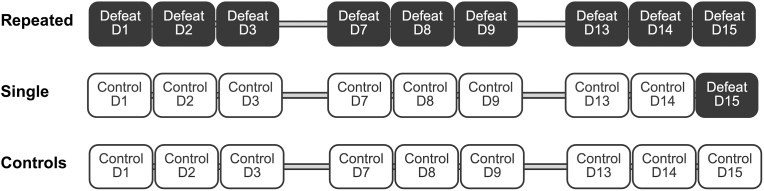
Schematic showing typical timeline of triads of daily defeat across 3 consecutive weeks in repeated defeat rats as contrasted with single defeat and control group rats. Latency to submission during social defeat episodes was measured. Subjects were euthanized 1 h or 6 h after the final session and central amygdala (CeA) punches were dissected for analysis of gene expression.

To investigate the effects of defeat on the expression of studied genes, experimental intruders were randomly assigned to 1 of 3 groups: control, single defeat, or repeated defeat (see Fig. 1). For defeat, females were removed and then intruders were introduced. Upon their submission, intruders were placed in a protective wire-mesh enclosure (20 × 20 × 32 cm) in the resident's home cage. Intruders remained in the enclosure for 30 min to receive further psychosocial threat. The wire enclosure prevented physical injury, but allowed auditory, olfactory, visual, and limited physical contact (mouth/nose). Repeated defeat rats were defeated 9 times across 15 days, involving 3 triads of 3 consecutive days of daily defeat. Triads were spaced by 3 non-test days. Single defeat rats were subjected to defeat only on the last day of defeat. For their other (non-defeat) days and on all test days for control group rats, subjects were handled and returned to their home cage without food for 30 min, corresponding to the duration of the “threat” period.

Subjects were euthanized by decapitation under rapid isoflurane anesthesia 1 h or 6 h after the final session. Central amygdala (CeA) punches were rapidly dissected on an ice-cold stage from 2-mm fresh coronal sections. Punches were stabilized with RNAlater (Qiagen Inc., Valencia, CA), maintained on dry ice until all dissections were complete, and then stored at −80 °C until qPCR, as described below.

### 2.3 Shock history ethanol reacquisition model

#### 2.3.1 Study 2 - gene expression cohort

Adult, male (*n* = 22) Wistar rats were individually housed for the reacquisition of EtOH self-administration model. Model details and effects of stress to increase EtOH reacquisition in this cohort have been reported previously (Logrip and Zorrilla, 2012). Briefly, rats were allowed 2-bottle choice (2BC) access to EtOH (10 % *v/v*) vs. water in their home cages for 48 h, followed by 4 days of daily, limited access (1 h/day) to 2BC EtOH, time-matched to future operant sessions. Rats then acquired operant water self-administration behavior via an overnight (16 h) fixed ratio 1 (FR1) session, with chow available *ad libitum,* during which responses at the active lever dispensed 0.1 mL of water. Responses at an inactive lever had no scheduled consequences. After 24 h, rats were pseudo-randomly assigned to Stress History (light cues co-terminating with 0.4-mA foot shocks) or Control groups (light cues alone), matched for home-cage EtOH intake and responses and preference for the active water lever. On 3 consecutive days, Stress subjects received daily 30-min sessions of cued (5-s house light) foot-shock stress (60 1-s 0.4-mA shocks/session, variable intershock-interval of mean 30-s; range 11–50 s). Controls received light cues only. Both light cue- and context-induced increases in immobility and freezing result from this procedure. Beginning 38–42 h later, rats acquired operant EtOH self-administration (FR1, 10 % *v/v*) during 5 1-h sessions conducted every 2–3 days, followed by 8 FR3 sessions. Lever pressing was then extinguished over 15 sessions (post-stress days 33–55), during which responses at the active lever had no scheduled consequences. On post-shock day 56, alcohol access was renewed for one session under a progressive ratio (PR) schedule (1,1,2,2,3,3,4,4,6,6,8,8,10,10,12,12,14,14,17,17, etc.) (Walker and Koob, 2007) followed by reacquisition of self-administration under an FR3 schedule (“relapse”). The mean intake during and blood EtOH concentration (BEC) after the first FR3 session were defined as relapse-like reacquisition (g/kg) and BEC (mg%), respectively. Forty-eight h after the last FR3 session, rats were decapitated under isoflurane anesthesia, and RNA-stabilized CeA punches were collected as described above.

#### 2.3.2 Study 3 - benztropine cohort

**Fig. 2:**
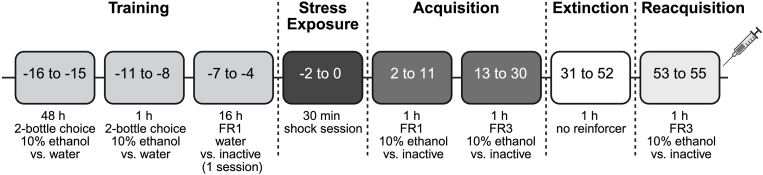
Timeline of behavioral testing in the foot shock + ethanol self-administration model. During operant ethanol self-administration sessions, number of active lever presses (ethanol responses/kg), ethanol intake (g/kg), and number of inactive lever presses were measured. Blood ethanol concentrations (BECs; mg%) were also obtained after the first FR3 reacquisition session. Forty-eight hours after the final FR3 reacquisition session, subjects were euthanized, and central amygdala (CeA) punches were collected for gene expression analysis.

As Fig. 2 shows, adult male and female Wistar rats (*n* = 20/sex) were prepared as described above, except that all rats received foot-shock stress exposure, 16 extinction sessions were performed, and no PR session was conducted. To assess the effects of benztropine on reacquisition of self-administration, rats were randomly assigned to repeated pretreatment (2 h) with 1 of 3 benztropine doses (0, 5, and 10 mg/kg) (Cruz et al., 2023) in a between-subjects design. Subjects received the same assigned dose before each of 3 reacquisition FR3 sessions.

### 2.4 RNA isolation, reverse transcription, and qPCR analysis – studies 1 and 2

RNA was isolated using QIAzol lysis reagent (Qiagen, Inc., Valencia, CA, USA) with shearing disruption using a discardable pestle and pestle motor per the manufacturer's instructions. RNA quantity and purity were determined via Nanodrop 2000c (Thermo Fisher Scientific, Waltham, MA, USA). Extracted total RNA was ezDNase-treated and reverse transcribed using SuperScript IV (SSIV) reverse transcriptase (RT) VILO kit (Thermo Fisher Scientific, Waltham, MA, USA) following the manufacturer's instructions. Gene expression levels were determined through a quantitative polymerase chain reaction (qPCR), performed using PowerTrack™ SYBR Green Master Mix (Thermo Fisher Scientific, Waltham, MA, USA), in a CFX 384 Real-Time System (Bio-Rad, Hercules, CA, USA) thermocycler. Primers were obtained from Integrated DNA Technologies (Coralville, IA, USA) (Table 1). The polymerase activation step was held for 3 min at 50 °C, followed by a denaturation step at 95 °C for 5 min, and then a 15-s denature step at 95 °C, followed by an annealing step for 15 s (temperature per the primer pair's *T*_*m*_), with the elongation step following at 72 °C for 15 s. The 15-s denaturation, annealing, and elongation steps were repeated 42 times. Melt curve analysis (95 °C for 15 s, then from 60 °C to 95.1 °C in 0.3 °C 1-min increments) was used to confirm a single PCR product at the expected temperature. The *ΔΔCt* method, utilizing *Gapdh* as the housekeeping reference gene, was used to calculate the relative fold change of gene expression.

**Table 1:** List of qPCR primers. Read from 5′ to 3’ left to right.

Transcript	Forward	Reverse
*Gapdh*	CAA CTC CCT CAA GAT TGT CAG CAA	GGC ATG GAC TGT GGT CAT GA
*Fkpb5*	GCC GGC AAG AAA CAC GAG AG	GAG GAG GGC CGA GTT CAT T
*Nr3c1a*	GCG ACA GAA GCA GTT GAG TCA TC	CCT TGC CTC CAC GTA ACT GTT AG
*Nr3c1b*	GCG CTT GAG GCT AAG ATA GCT	CCC ATG TTT CTG CCT CTT TCT TTG
*Chrm1*	TCC CTC ACA TCC TCC GAA GGT G	CTT TCT TGG TGG GCC TCT TGA CTG
*Chrm3*	ACC ACG GCT ACT CTA CCT CTG TCC TTC A	AGC GTC TGG GCG GCC TTC TTC TC
*Hrh1*	CTTCTCCTTCCTGTGGGTTATAC	AGTCTGTCTCACACTTGTCTTC
*Fos*	CAG CCT TTC CTA CTA CCA TCC C	ACA GAT CTG CGC AAA AGT CC
*Fosb*	GTG AGA GAT TTG CCA GGG TC	AGA GAG AAG CCG TCA GGT TG
*ΔFosb*	AGG CAG AGC TGG AGT CGG AGA T	GCC GAG GAC TTG AAC TTC ACT CG
*Egr1*	TGC ACC CAC CTT TCC TAC TC	AGG TCT CCC TGT TGT TGT GG
*Egr2*	CAA GGC CGT AGA CAA AAT CCC A	CCC ATG TAA GTG AAG GTC TGG T
*Junb*	TCT TTC TCT TCA CGA CTA CA	CTA GCT TCA GAG ATG CG
*Jun*	TCT CAG GAG CGG ATC AA	TGT TAA CGT GGT TCA TGA C

Table 1 shows a list of the primers used to quantify transcripts for the reference gene encoding Glyceraldehyde-3-phosphate dehydrogenase (*Gapdh*) and the target genes encoding FK506-binding protein 51 *(Fkbp5;* nomenclature for the rat ortholog per Rat Genome Database*),* GR*α* and GR*β* (*Nr3c1a, Nr3c1b*), and the IEG products Protein c-Fos (*Fos*), Protein Fos B (*Fosb*) and its truncated variant ΔFosb (*ΔFosb),* early growth response protein 1 (*Egr1*)*,* early growth response protein 2 (*Egr2*)*,* Transcription factor Jun (*Jun*)*,* and Transcription factor Jun B (*Junb)*. Because of benztropine's pharmacological activity at other targets and to determine the specificity of CeA changes in *Fkbp5*, we also measured potential changes in gene expression of M_1_ and M_3_ muscarinic acetylcholine (*Chrm1, Chrm3*) and histaminergic H_1_ (*Hrh1*) receptors, for which benztropine also has affinity.

### 2.5 Statistical analysis

Gene expression analysis was performed using *z*-scores of the ΔΔCt's multiplied by −1 to represent directionality, standardized to values of unstressed controls. Potential outliers were defined a) using Dixon's *Q*-test on raw Ct data and b) as *z*-scored values with high studentized residual scores (≥|3.0|) that had undue leverage (>2-fold the jackknifed mean leverage of other samples) (Cook and Weisberg, 1982) or influence (Cook's *D* > the 50 %ile of the *F*-distribution, *F*[Cook's *D*](*k*+1,*n*-*k*-1), where *k* = number of predictor variables and *n* = total samples) (Cook and Weisberg, 1982). To maintain constant sample size and facilitate correlation analyses, outliers and missing values (1 of 192 1-h post-defeat z-scores, 0.52 % of data) were replaced using multiple imputation (Van Ginkel et al., 2020) as the average of 10 independent estimates in SPSS. Gene expression data for the social defeat model were analyzed separately for the animals euthanized 1 h vs. 6 h after the last defeat session by Generalized Linear Model analysis with Wald Chi-square that more appropriately and powerfully model non-normally distributed data and are more robust in small sample contexts (Nelder and Wedderburn, 1972; Stroup, 2015) with Defeat Type as a between-subject factor. Following significant tests, post hoc pairwise comparisons used LSD tests for data with equal variance.

Rats in the reacquisition gene expression study were defined as showing high or low self-administration (reacquisition session intake >0.5 g/kg = high (Logrip and Zorrilla, 2012);) and pharmacologically relevant (BEC >25 mg%) (or not) BECs during the FR3 reacquisition session. Gene expression data were analyzed by Generalized Linear Model analysis with Wald Chi-square, comparing: 1) shocked vs. control, 2) high vs. low reacquisition intake, and 3) pharmacologically relevant vs. low reacquisition BEC. Pearson correlations were used to relate gene expression results from both behavior models to other transcripts and behaviors (average defeat latency from all 9 sessions, reacquisition intake, BECs).

Data from the benztropine-shock history reacquisition drinking study were analyzed using 3-way ANOVA; Dose and Sex were between-subject factors and Day a within-subject factor. Linear and quadratic Dose contrasts were performed to identify dose-related effects. Following significant ANOVA effects, post hoc LSD tests were performed to identify significant pairwise differences. To interpret effects further, 2-way ANOVA (Dose and Day effects) also was performed on subjects separated by sex. Statistical significance was defined as *α* = 0.05.

## 3 Results

### 3.1 Study 1 - defeat stress, CeA IEG and *Fkbp5* expression, and submission latency

#### 3.1.1 IEG expression at 1 h post-defeat

**Fig. 3:**
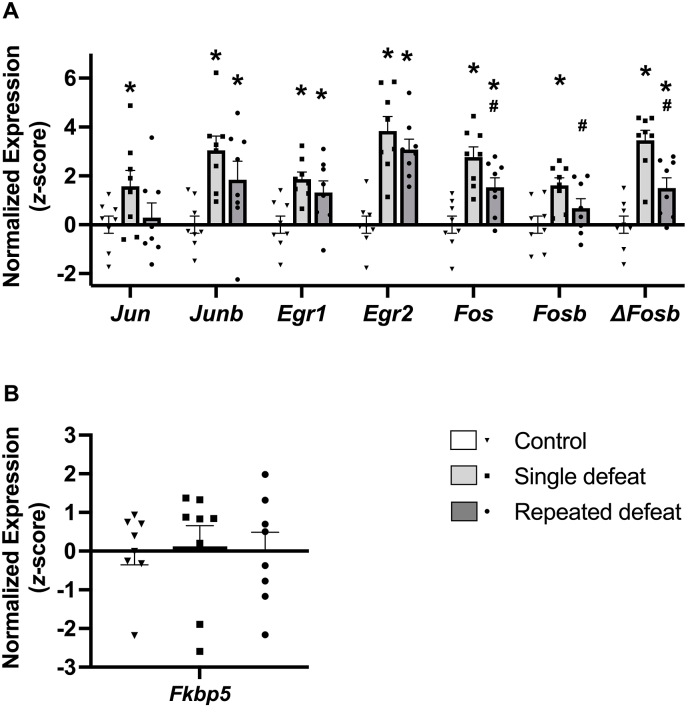
A) Effects of single and repeated defeat on expression of the genes encoding transcription factor Jun (*Jun*), transcription factor Jun B (*Junb*), early growth response protein 1 (*Egr1*), early growth response protein 2 (*Egr2*), protein c-Fos (*Fos*), protein FosB (*FosB*), and truncated protein *Δ*FosB (*ΔFosB*) in the central nucleus of the amygdala (CeA) of male rats 1 h after the last defeat session. Each bar represents the mean z-score ± standard error, standardized to unstressed controls, with shape scatter showing individual values. *n* = 8/type of defeat paradigm. LSD test after significant omnibus test. ∗*p* < 0.05 vs. unstressed controls, #*p* < 0.05 vs. single defeat. B) Effects of single and repeated defeat on the expression of the gene encoding FKBP prolyl isomerase 5 (*Fkbp5*) in the CeA of male rats 1 h after the last defeat session. Gene expression for all panels is expressed as z-scores, standardized to unstressed controls. Each bar represents the mean z-score ± standard error, showing individual values. *n* = 8/type of defeat paradigm.

As expected and shown in Fig. 3, defeat significantly increased CeA mRNA expression of all IEGs at 1 h post-defeat (*Jun*: Wald *X*^2^_*3*_ *= 9.58, p = 0.02; Junb*: Wald *X*^2^_*3*_ *= 41.78, p < 0.001; Egr1*: Wald *X*^2^_*3*_ *= 41.56, p < 0.001*; *Egr2*: Wald *X*^2^_*3*_ *= 125.63, p < 0.001*; *Fos*: Wald *X*^2^_*3*_ *= 76.71, p < 0.001*; *Fosb*: Wald *X*^2^_*3*_ *= 28.32, p < 0.001*; Δ*Fosb*: Wald *X*^2^_*3*_ *= 103.73, p < 0.001*). Specifically, single defeat significantly increased the expression of *Jun* (*p* = 0.03), *Junb* (*p* < 0.001), *Egr1* (*p* < 0.001), *Egr2* (*p* < 0.001), *Fos* (*p* < 0.001), *Fosb* (*p* = 0.001), and Δ*Fosb* (*p* < 0.001) compared to unstressed controls. At 1 h post-single defeat, *Egr2* and *ΔFosb* showed the greatest levels of mRNA expression vs. unstressed controls (+3.8 and 3.5 *z*-score units, respectively; Fig. 3). Repeated defeat also still significantly increased expression of *Junb* (*p* = 0.018), *Egr1* (*p* = 0.009), *Egr2* (*p* < 0.001), *Fos* (*p* = 0.003), and *ΔFosb* (*p* = 0.004) compared to unstressed controls, with greatest effect again for *Egr2* (+3.1 z-score units). However*,* for *Fos* (*p* = 0.02), *Fosb* (*p* = 0.045) and *ΔFosb* (*p* < 0.001), the increase in IEG expression of rats that underwent repeated defeat was significantly smaller than for single defeat.

Similarly, for all 7 IEGs, expression means were descriptively lower in the repeated defeat group compared to the single defeat group. The pattern of all 7 means being descriptively lower is unlikely to occur by chance, per a binomial distribution test (*p* < 0.008), suggesting an adaptive response in the repeated defeat rats.

**Supplemental Fig. 1:**
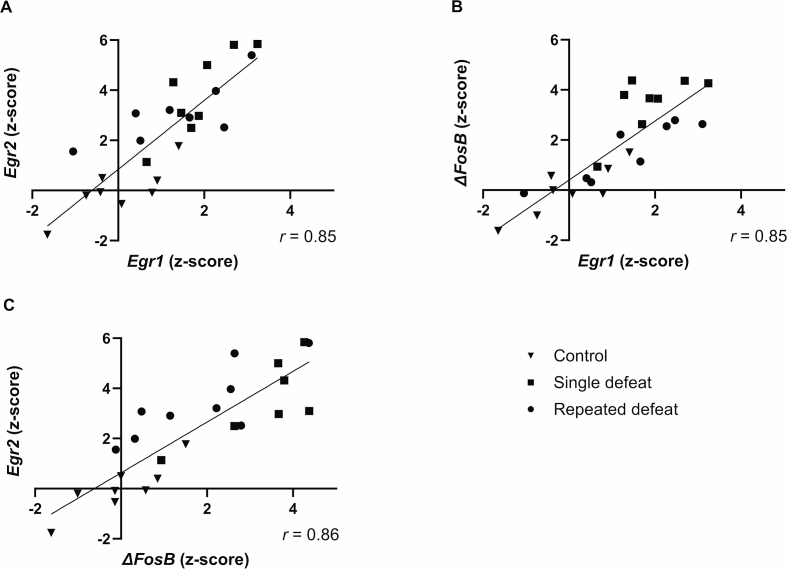
Scatterplot and Pearson correlations of gene expression of A) early growth response protein 1 (*Egr1*) with early growth response protein 2 (*Egr2*), B) *Egr1* with truncated protein *Δ*FosB (*ΔFosB*), and C) *Egr2* with *ΔFosB* in the central nucleus of the amygdala (CeA) of male rats euthanized 1 h after unstressed control (triangles), single defeat (squares), or repeated defeat (circles) conditions. All *p*s < 0.05. Gene expression is expressed as z-scores, standardized to unstressed controls *n* = 24.

Expression of all IEGs at 1 h correlated with one another (*r*s = 0.57–0.86, *p*s < 0.005), with strongest intercorrelations for *Egr1*, *Egr2*, and *ΔFosb* (*r*s = 0.85–0.86, *p*s < 0.0001) (Supplemental Fig. 1).

#### 3.1.2 IEGs and Fkpb5 at 1 h post-defeat: relation to submission latency

**Fig. 4:**
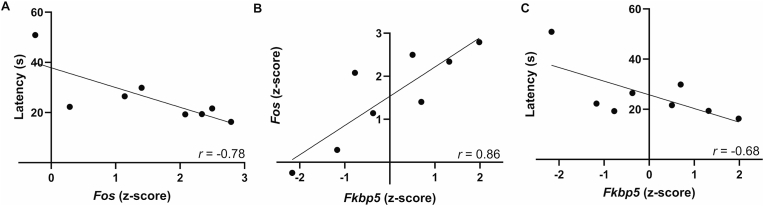
Scatterplot and Pearson correlations between A) average repeated submission latency and central nucleus of the amygdala (CeA) protein c-Fos (*Fos*) expression, B) CeA *Fos* and FKBP prolyl isomerase 5 (*Fkbp5*) gene expression, and C) average repeated submission latency and CeA *Fkbp5* expression, in repeated defeat rats euthanized 1 h after the last defeat session. Gene expression is expressed as z-scores, standardized to unstressed controls, *n* = 8, all *p*s < 0.05.

**Supplemental Fig. 2:**
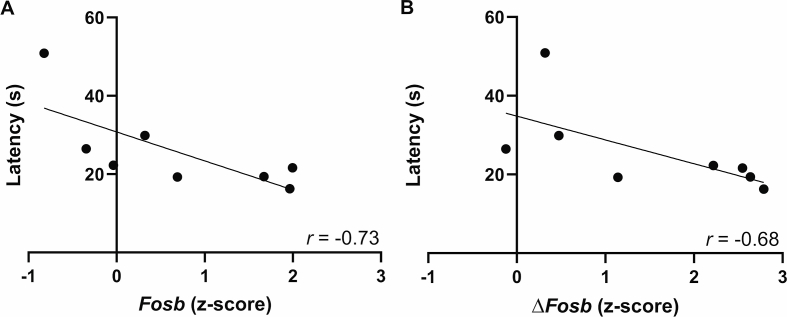
Scatterplot and Pearson correlations between average repeated submission latency and CeA A) *Fosb* (*p* < 0.04) or B) *ΔFosb* expression (*p* < 0.08) in repeated defeat rats euthanized 1 h after the last defeat session. Gene expression is expressed as z-scores, standardized to unstressed controls *n* = 8.

As Fig. 4A shows, repeated defeat rats that had briefer average latencies to submit showed greater CeA *Fos* expression at 1 h post-defeat, resulting in a significant inverse correlation (*r*(8) *=* −0.78*, p* = 0.02, Fig. 4A). Similar inverse correlations were seen of average submission latency to *Fosb*. (*r*(8) *=* −0.73*, p* = 0.04) and Δ*Fosb* expression (r(8) = −0.65, *p* = 0.08) (Supplemental Fig. 2). In single defeat rats, there was no significant relation of *Fos* (r(8) = −0.16), *Fosb* (r(8) = −0.03), or Δ*Fosb* (r(8) = −0.26) to defeat latency, indicating that the relations were specific to repeated defeat.

**Supplemental Fig. 3:**
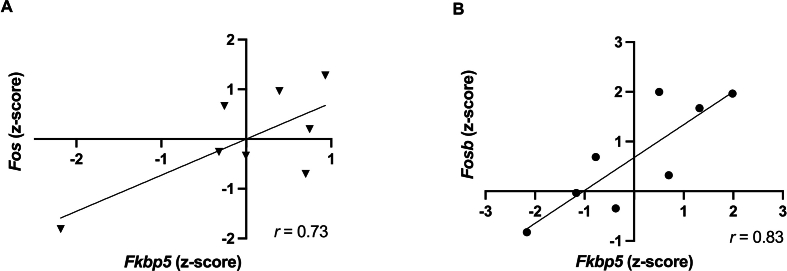
A) Scatterplot and Pearson correlation between *Fkbp5* and *Fos* expression in control group rats sacrificed 1 h after the final control procedure, *p* < 0.05. B) Scatterplot and Pearson correlation between *Fkbp5* and *FosB* expression in repeated defeat rats sacrificed 1 h after the final defeat, *p* < 0.05.

Consistent with it not being an IEG, mean CeA levels of *Fkbp5* were not altered by 1 h after defeat (Fig. 3B). As Fig. 4B shows, however, repeated defeat rats that had greater CeA *Fkbp5* expression also showed significantly greater CeA *Fos* at 1 h post-defeat (*r*(8) *=* 0.86*, p* = 0.007). *Fkbp5* also correlated directly with CeA *Fos* expression in unstressed controls (*r*(8) = 0.73, *p* = 0.04) (Supplemental Fig.3A).

In addition to *Fos*, *Fkbp5* also correlated significantly with *Fosb* (*r*(8) = 0.83, *p* = 0.01) in repeated defeat rats (Supplemental Fig. 3B), but not with *Egr1* (*r*(8) = 0.54, *p* = 0.167), *Egr2* (*r*(8) = 0.22, *p* = 0.596), or Δ*FosB* (*r*(8) = 0.57, *p* = 0.14). As shown in Fig. 4C, greater CeA *Fkbp5* expression in repeated defeat rats correlated with briefer average submission latencies (*r*(8) = −0.68, 1-tailed *p* = 0.03).

#### 3.1.3 Fkbp5 and GR expression at 6 h post-defeat

**Fig. 5:**
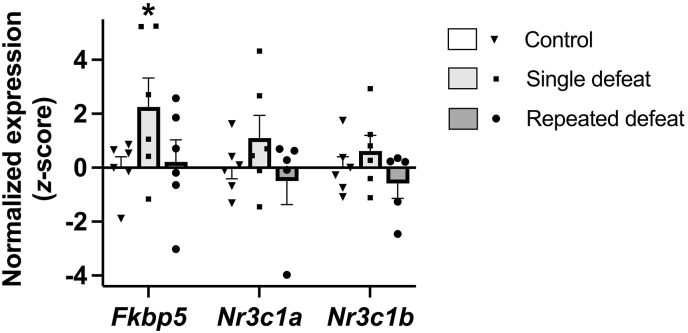
Effects of single and repeated defeat on the expression of the gene encoding FKBP prolyl isomerase 5 (*Fkbp5*), canonical glucocorticoid receptor α (*Nr3c1a*), and truncated glucocorticoid receptor β (*Nr3c1b*) in the central nucleus of the amygdala (CeA) of male rats 6 h after the last defeat session. Gene expression is expressed as z-scores, standardized to unstressed controls. Each bar represents the mean z-score ± standard error, with shape scatter showing individual values. *n* = 5–6/type of defeat paradigm. ∗*p* < 0.05 vs. unstressed controls.

By 6 h post-defeat, as Fig. 5 shows, defeat significantly increased CeA mRNA expression of *Fkbp5* (Wald *X*^2^_*3*_ = 9.29, *p* = 0.026). Specifically, single defeat (*p* = 0.032), but not repeated defeat, increased *Fkpb5* expression relative to unstressed controls. In contrast, there were no significant effects of social defeat on the mean CeA expression of the GR-encoding mRNA transcripts *Nr3c1a* (Wald *X*^2^_*3*_ = 3.41, *p* = 0.33) and *Nr3c1b* (Wald *X*^2^_*3*_ = 3.24, *p* = 0.36) at 6 h post-defeat (Fig. 5).

**Supplemental Fig. 4:**
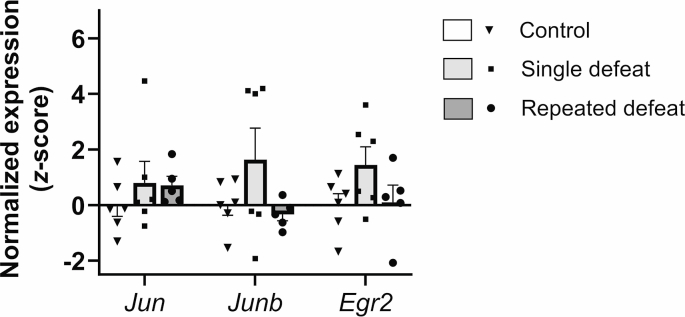
Effects of single and repeated defeat on expression of the genes encoding transcription factor Jun (*Jun*), transcription factor Jun B (*Junb*), and early growth response protein 2 (*Egr2*) in the central nucleus of the amygdala (CeA) of male rats 6 h after the last defeat session. Each bar represents the mean z-score ± standard error, standardized to unstressed controls, with scatter for individual values. *n* = 5–6/type of defeat paradigm.

As expected for the transient time course of IEGs, a subset of IEGs that were measured at 6 h post-defeat (*Jun, Junb, Egr2*) showed descriptively reduced mean expression as compared to 1 h post-defeat. However, none of these descriptive differences were statistically significant with inferential testing (see Supplemental Fig. 4), so they were not studied further.

### 3.2 Study 2 - foot-shock stress, CeA IEG, *Fkbp5*, and GR expression, and EtOH relapse-like reacquisition

#### 3.2.1 Foot-shock stress vs. unstressed controls

**Supplemental Fig. 5:**
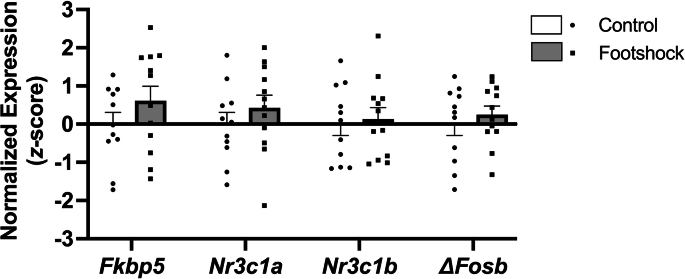
Gene expression of FKBP prolyl isomerase 5 (*Fkbp5*), canonical glucocorticoid receptor α (*Nr3c1a*), truncated glucocorticoid receptor β (*Nr3c1b*), and truncated protein *Δ*FosB (*ΔFosB*) in the central nucleus of the amygdala (CeA) of male rats that underwent a repeated foot shock/EtOH self-administration model. Gene expression is expressed as z-scores, standardized to unstressed controls. Each bar represents the mean *z*-score ± standard error, showing individual values. *n* = 11–12/foot shock experience.

**Supplemental Fig. 6:**
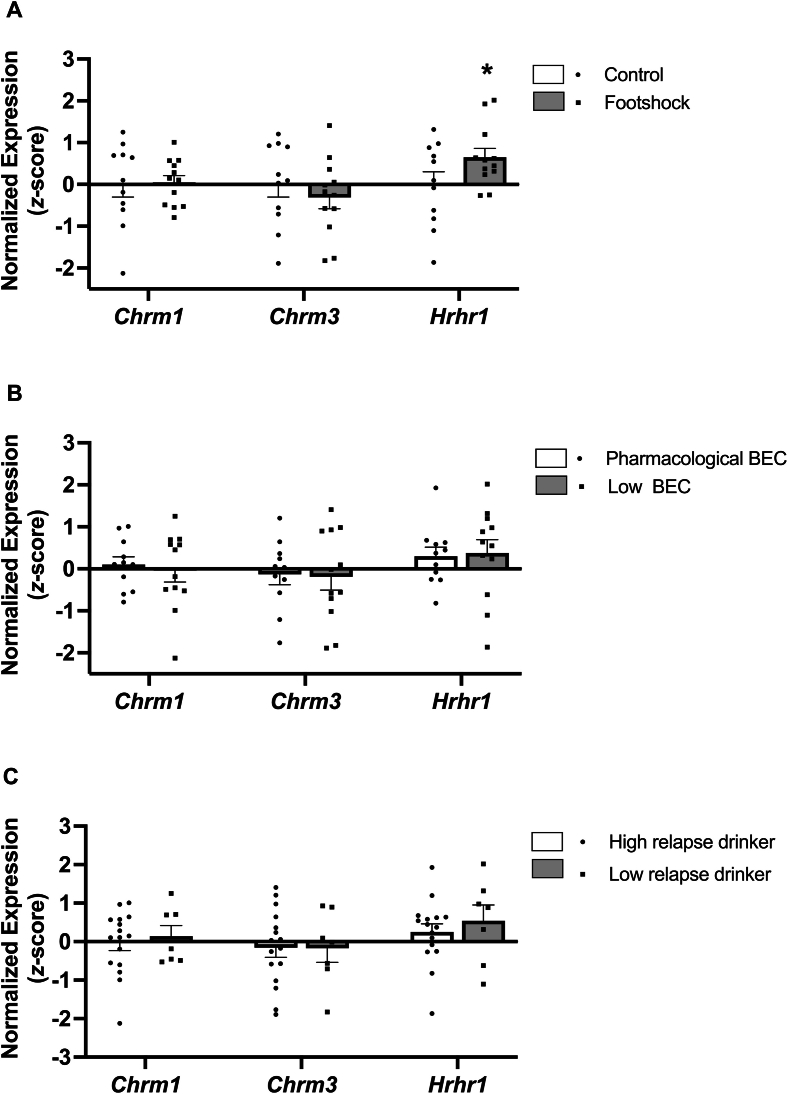
Gene expression of muscarinic acetylcholine receptor 1 (*Crhm1*), muscarinic acetylcholine receptor 3 (*Crhm3*), and histamine receptor H_1_ (*Hrh1*) in the central nucleus of the amygdala (CeA) of male rats that underwent a repeated foot shock/EtOH self-administration model. Panels show expression in relation to A) foot-shock history, B) blood ethanol concentrations (BEC), or C) ethanol intake associated with relapse-like reacquisition of ethanol self-administration. Gene expression is expressed as z-scores, standardized to unstressed controls. Threshold of pharmacologically relevant BEC = 25 mg%. Threshold of “high” relapse-like drinking = 0.5 g/kg EtOH (Logrip and Zorrilla, 2012). Each bar represents the mean *z*-score ± standard error, showing individual values (B) *n* = 11–12/BEC level, (C) *n* = 7–16/drinking level. ∗*p* < 0.05.

In the reacquisition of EtOH self-administration model, rats with a history of foot-shock stress did not show differences in mean CeA expression of Δ*FosB, Fkbp5*, or GR transcripts at study end (∼2 months post-shock) vs. unstressed controls (Supplemental Fig. 5). However, CeA gene expression of H_1_ histaminergic receptor encoding gene differed according to foot-shock history (Wald *X*^2^_2_ = 7.43, *p* = 0.024), as shown in Supplemental Fig. 6.

#### 3.2.2 Pharmacologically relevant BECs vs. low BECs

**Fig. 6:**
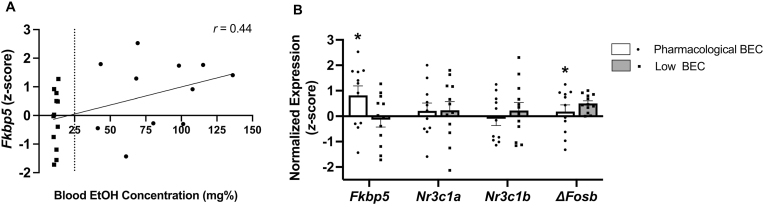
(A) Correlation of FKBP prolyl isomerase 5 (*Fkbp5*) gene expression in the central nucleus of the amygdala (CeA) vs. blood ethanol concentration (BEC) during the first FR3 relapse-like reacquisition session of male rats that underwent a repeated foot shock/EtOH self-administration model. (B) Gene expression of *Fkbp5*, canonical glucocorticoid receptor α (*Nr3c1a*), truncated glucocorticoid receptor β (*Nr3c1b*), and truncated protein *Δ*FosB (*ΔFosB*) in the CeA of male rats that underwent a repeated foot shock/EtOH self-administration model. Gene expression is expressed as z-scores, standardized to unstressed controls. The pharmacologically relevant threshold of BEC was defined as 25 mg% EtOH. Each bar represents the mean *z*-score and standard error, with shape scatter showing individual values. *n* = 11–12/BEC level. ∗*p* < 0.05.

A direct correlation was found between CeA expression of *Fkbp5* and BEC on the first “relapse” reacquisition day (*r*(23) = 0.44, *p* = 0.03, Fig. 6A). Accordingly, rats with pharmacologically relevant BECs (>25 mg%) showed higher *Fkbp5* expression as compared to those with lower BECs (Wald *X*^2^_2_ = 6.65, *p* = 0.036), Fig. 6B). Moreover, Δ*FosB* expression levels were lower in rats with pharmacologically relevant BECs relative to low BECs (Wald *X*^2^_2_ = 8.81, *p* = 0.012). Analysis did not find differences between pharmacologically relevant vs. low-BEC groups for GR (Fig. 6B), M_1_, M_3_, and H_1_ (Supplemental Fig. 6) transcripts.

#### 3.2.3 High drinkers vs. low drinkers

**Fig. 7:**
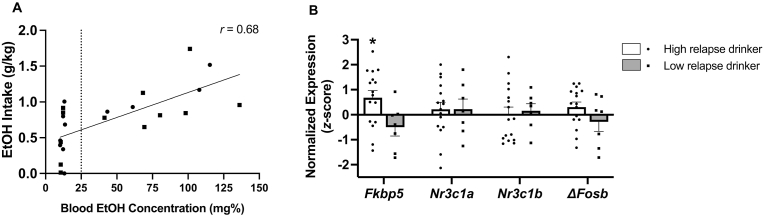
(A) Pearson correlation of 2-h 2-bottle choice ethanol intake with blood ethanol concentration (BEC) during the first FR3 relapse-like reacquisition session of male rats that underwent a repeated foot shock/EtOH self-administration model, ∗*p* < 0.001. (B) Gene expression of FKBP prolyl isomerase 5 (*Fkbp5*), canonical glucocorticoid receptor α (*Nr3c1a*), truncated glucocorticoid receptor β (*Nr3c1b*), and truncated protein *Δ*FosB (*ΔFosB*) in the central nucleus of the amygdala (CeA) of male rats that underwent a repeated foot shock/EtOH self-administration model. The threshold of “high” relapse-like drinking was defined as 0.5 g/kg EtOH (Logrip and Zorrilla, 2012). Gene expression is expressed as z-scores, standardized to unstressed controls. Each bar represents the mean *z*-score and standard error, with shapes showing individual values. *n* = 7–16/drinking level. ∗*p* < 0.05.

As expected, EtOH self-administration during the first reacquisition day correlated significantly with BEC at session end (*r*(23) = 0.68, *p* < 0.001, Fig. 7A). Accordingly, as Fig. 7B shows, rats with high EtOH self-administration (>0.5 g/kg) on the “relapse” reacquisition day also showed significantly higher CeA *Fkbp5* mRNA expression than rats with low “relapse” EtOH self-administration (Wald *X*^2^_2_ = 8.50, *p* = 0.014). CeA gene expression of M_1_ and M_3_ acetylcholine receptors and H_1_ histaminergic receptors, other targets of benztropine action, did not differ by stress history, reacquisition BEC, or high EtOH self-administration on the “relapse” reacquisition day (Supplemental Fig. 6).

### 3.3 Study 3 - benztropine effect on EtOH reacquisition

#### 3.3.1 EtOH self-administration responses

**Fig. 8:**
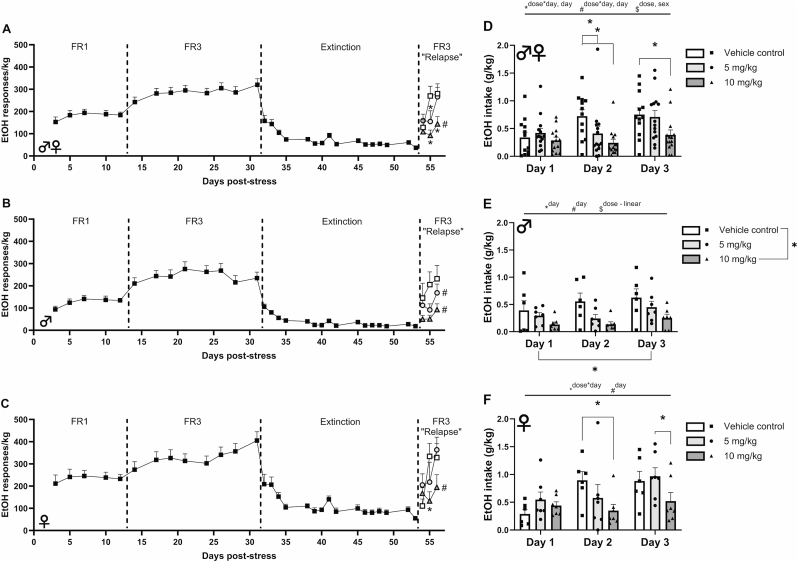
Effects of benztropine mesylate pretreatment (−120 min) on weight-normalized EtOH responses (responses/kg) (left panels) and total EtOH intake (g/kg, right panels) in (A,D) male + female, (B,E) male, and (C,F) female rats during 2-lever EtOH-reinforced vs. inactive lever self-administration. Left panel line graphs (A,B,C) show average EtOH responses per kg body weight. Right panel histograms (D,E,F) show group mean (*M*) + standard error (SEM), and shapes show the scores of individual subjects. Rats received no pharmacological treatment before the “relapse” phase, at which time they were assigned to 1 of 3 repeated systemic treatment doses (i.p., vehicle, 5, or 10 mg/kg). *M* + SEM. *n* = 10–14/sex. Individual drug-treated conditions marked by the asterisk differ from their respective vehicle on that day. Individual conditions in the left panels marked by the pound (#) sign differ from their respective pre-extinction average (last 3 days of baseline FR3 sessions). ∗ANOVA within-subject effect. ^#^ANOVA within-subject contrast. ^$^ANOVA between-subject effect, *p* < 0.05.

Fig. 8 displays the number of presses on the alcohol-reinforced lever, normalized to the rats' body weight, for all rats (Fig. 8A), males alone (Fig. 8B), and females alone (Fig. 8C). These data, prior to benztropine treatment, show similar behavioral patterns between sexes during initial FR1 and FR3 acquisition as well as extinction. Due to previously reported sex differences in FKBP51 inhibitor action, potential benztropine effects were analyzed for all subjects and also separately for males and females.

As Fig. 8A (right side) also shows, analyses of weight-normalized EtOH responding (responses/kg) during reacquisition showed benztropine Dose (*F*_*2, 33*_ = 5.68, *p* = 0.008) and Dose × Day effects (*F*_*4, 66*_ = 3.18, *p* = 0.02) in rats with renewed EtOH access after extinction. Pairwise comparisons showed that 10 mg/kg benztropine significantly reduced EtOH responses vs. vehicle on days 2 (*p* = 0.001) and 3 (*p* = 0.009). The 5 mg/kg benztropine dose also decreased EtOH responses vs. vehicle on day 2 (*p* = 0.02). A Sex main effect (*F*_*1, 33*_ = 5.26, *p* = 0.03) reflected that female rats showed greater weight-normalized responding for EtOH than males. Day main (*F*_*2, 66*_ = 3.65, *p* = 0.03) and linear contrast effects (*F*_*1, 33*_ = 7.93, *p* = 0.008) reflected that EtOH responding (responses/kg) increased with repeated days of renewed access (Fig. 8A).

Likewise, in males analyzed separately, benztropine pretreatment yielded a significant main effect of Dose (*F*_*2, 16*_ = 9.06, *p* = 0.002, Fig. 8B) on EtOH responding, reflecting that 10 mg/kg benztropine significantly decreased EtOH responses vs. vehicle control (*p* = 0.007), independent of the injection day. In females analyzed separately, a significant Dose × Day interaction (*F*_*4, 32*_ = 2.77, *p* = 0.044, Fig. 8C) was observed on EtOH responses. Pairwise comparisons showed that 10 mg/kg benztropine decreased weight-normalized EtOH responding on day 2 vs. vehicle (*p* = 0.01) and on day 3 vs. the lower benztropine dose (*p* = 0.02) (*p* = 0.07 vs. vehicle).

#### 3.3.2 Comparison to pre-extinction levels

To determine the effects of benztropine on reacquisition, the weight-normalized level of EtOH self-administration attained by the last day of reacquisition was compared to the pre-extinction baseline, defined as the average of weight-normalized lever presses on the last 3 days of FR3 acquisition. A significant Dose × Day interaction (*F*_*2, 34*_ = 3.81, *p* = 0.03) as well as main effects of Day (*F*_*1, 34*_ = 6.99, *p* = 0.01) and Sex (*F*_*1, 34*_ = 17.09, *p* < 0.001) were observed. Pairwise comparisons showed that rats treated with 10 mg/kg of benztropine during reacquisition pressed significantly less for EtOH than they had during the pre-extinction baseline (*p* = 0.001, Fig. 8A). In contrast, a significant reduction was not seen for rats treated with the 5 mg/kg dose (*p* = 0.15) or vehicle (*p* = 0.71).

When we analyzed the males separately, a similar Dose × Day interaction (*F*_*2, 17*_ = 8.14, *p* = 0.003) was observed, as well as a main effect of Day (*F*_*1, 17*_ = 8.71, *p* = 0.009). Pairwise comparisons showed that male rats treated not only with the 10 mg/kg dose of benztropine (*p* = 0.03), but also the 5 mg/kg dose (*p* = 0.04), showed significantly reduced lever pressing for EtOH during reacquisition as compared to their pre-extinction baseline levels (Fig. 8B). In contrast, in female rats, only the 10 mg/kg dose of benztropine significantly reduced weight-normalized lever presses on the last day of reacquisition as compared to pre-extinction levels (*p* = 0.007, Fig. 8C).

#### 3.3.3 Inactive responses

**Supplemental Fig. 7:**
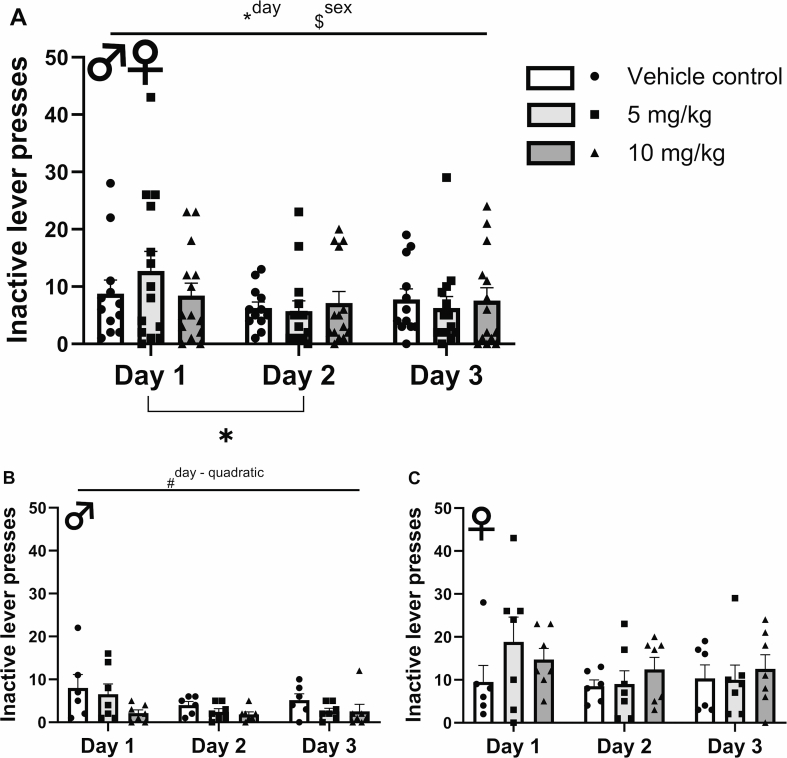
Effects of benztropine mesylate pretreatment (−120 min) on inactive lever presses in (A) male + female, (B) male, and (C) female rats during 2-lever EtOH-reinforced vs. inactive self-administration. Histograms show group mean + standard error , and shapes show the scores of individual subjects. *n* = 10–14/sex; there were no significant Dose or Dose X Day effects on inactive lever responding.

As shown in Supplemental Fig. 7, there were no significant Dose or Dose × Day effects of benztropine on inactive responses considering all subjects together or males and females separately. A Sex main effect (*F*_*1, 34*_ = 21.71, *p* < 0.001) reflected that female rats had higher inactive responses than males. Main (*F*_*2, 68*_ = 4.54, *p* = 0.01) and quadratic effects of Day (*F*_*1, 34*_ = 6.79, *p* = 0.01) reflected that rats pressed the inactive lever more on the first than the 2nd day of reacquisition.

#### 3.3.4 EtOH self-administration intake

In terms of EtOH intake, Fig. 8D shows that benztropine pretreatment yielded significant Dose (*F*_*2, 34*_ = 4.397, *p* = 0.02) and Dose × Day effects (*F*_*4, 68*_ = 3.568, *p* = 0.011, Dose × Day quadratic contrast (*F*_*2, 34*_ = 4.162, *p* = 0.024) on self-administered EtOH (g/kg) in male and female rats with renewed EtOH access after extinction. Pairwise comparisons to interpret the Dose × Day interaction showed that 10 mg/kg benztropine significantly reduced intake vs. vehicle on days 2 (*p* = 0.002) and 3 (*p* = 0.013, Fig. 8D). On day 2, the 5 mg/kg benztropine dose also decreased EtOH intake (*p* = 0.021, Fig. 8D). A significant Sex main effect (*F*_*2, 34*_ = 2.121, *p* = 0.004) reflected that female rats had higher reacquisition of EtOH self-administration (g/kg) than males. Main (*F*_*2, 68*_ = 11.806, *p* < 0.001) and linear contrast (*F*_*1, 34*_ = 24.659, *p* < 0.001) effects of Day reflected that EtOH self-administration (g/kg) increased with repeated days of renewed access (Fig. 8D).

As Fig. 8E shows for males, benztropine pretreatment yielded a significant main effect of Dose (*F*_*2, 17*_ = 3.78, *p* = 0.044), reflecting that 10 mg/kg benztropine significantly decreased EtOH intake vs. vehicle control (*p* = 0.025), independent of the injection day, in male rats. Main (*F*_*2, 34*_ = 5.28, *p* = 0.01) and linear contrast Day effects (*F*_*1, 17*_ = 10.93, *p* = 0.004) reflected that male rats self-administered significantly more EtOH on day 3 than day 1 of reacquisition (*p* = 0.03), independent of treatment. In females, unlike males, a significant Dose × Day interaction (*F*_*4, 34*_ = 2.91, *p* = 0.04) was observed. As Fig. 8F shows, pairwise comparisons showed that 10 mg/kg benztropine decreased EtOH self-administration on day 2 vs. vehicle (*p* = 0.013) and on day 3 vs. the lower benztropine dose (*p* = 0.025), without significantly reducing intake on day 1. A Day main effect (*F*_*2, 34*_ = 7.21, *p* = 0.002) reflected that EtOH self-administration increased across days, as expected.

## 4 Discussion

### 4.1 Overview

The present gene expression and pharmacological results are consistent with the hypotheses that *Fkbp5* expression or FKBP51 activity is associated with passive stress-coping behaviors and that the CeA may serve as a key hub for these effects. Acute resident-intruder stress, which promotes subordination, rapidly increased CeA neuronal activation in male rats, as indicated by elevated expression of IEGs, and enhanced CeA *Fkbp5* expression by 6 h post-defeat. Repeated defeat induced less IEG activation than single defeat, but continued *Fos* expression occurred in strong relation to *Fkbp5* expression. Both *Fkbp5* and *Fos* expression in the CeA correlated with faster submission across repeated defeat experiences, a passive response to social threat. In another model of passive stress coping, greater CeA *Fkbp5* expression was associated with greater EtOH intake and attainment of higher BECs during relapse-like reacquisition of EtOH self-administration. Finally, benztropine, which inhibits the association of FKBP51 with GR (Sabbagh et al., 2018), dose-dependently reduced relapse-like EtOH intake in the reacquisition model, with more robust actions in male than female rats. Thus, CeA *Fkbp5* expression correlated with post-stress CeA neuronal activation and two passive stress-coping behaviors – social submission and relapse-like EtOH self-administration. Further, benztropine, an FDA-approved, non-selective FKBP51 inhibitor with repurposing potential, reduced the reacquisition of EtOH self-administration in a model of post-traumatic relapse drinking. The latter result complements our recent finding that the FKBP51 inhibitors benztropine and SAFit-2 reduced voluntary EtOH intake in a comorbid PTSD/AUD model (Cruz et al., 2023).

Here, we did not assess the causal role of CeA Fkbp5 expression in mediating CeA IEGs’ responses to stress, social submission, reacquisition of EtOH self-administration, or the benztropine treatment effects; these remain to be determined. The present findings warrant causal study of CeA FKBP51 in passive stress coping and evaluation of selective FKBP51 inhibitors to reduce EtOH relapse-like behavior following a history of repeated stress.

### 4.2 Study 1 - social defeat, CeA IEG induction, and submission

Social defeat acutely increased the expression of all studied IEGs in the CeA within 1 h of the resident-intruder session. This included *Jun*, *Junb*, *Egr1*, *Egr2*, *Fos*, *FosB*, and *ΔFosB*. The largest effect size increases were seen for *Egr2* and *ΔFosB*. The latter finding at first may seem counterintuitive since ΔFosB protein is known as a long-term and enduring, rather than rapid, indicator of neuronal activation in medium spiny neurons (MSNs) (McClung et al., 2004). However, ΔFosB accumulation is cell-type- and region-specific; studies reported it accumulated differentially in dynorphin-positive striatal MSNs post-cocaine or stress and not as markedly in CeA neurons (Moratalla et al., 1996; Nye et al., 1995; Perrotti et al., 2004). Further, the long (8–10 h) accumulating half-life of ΔFosB results from protein, rather than mRNA, regulation – specifically, absence of C-terminal degradation signals in the truncated protein as well as post-translational phosphorylation by casein kinase 2 that converts it to its stable 37 kDa form (Carle et al., 2007; Ulery et al., 2006). In contrast to the protein, ΔFosB mRNA decays just as fast as mRNAs for other IEGs (half-life∼4 min) (Jurado et al., 2007). The mRNA was reportedly abundantly induced more after acute than repeated stimulation (Alibhai et al., 2007; Jurado et al., 2007; Zhang et al., 2014), similar to our findings.

Like early studies with hamsters (Kollack-Walker et al., 1997, 1999) and rats (Martinez et al., 1998), IEG induction was substantially blunted after a 9th (repeated) defeat, indicating neuroadaptation. Briefer subordination latencies in repeatedly stressed rats correlated strongly with greater CeA *Fos* expression. Similarly, in a previous study, rodents that demonstrated “low fight,” defined as submitting more quickly, had significantly more Fos-positive neurons in the amygdala when compared to control animals exposed to an empty resident's cage (Walker et al., 2009).

The positive correlations observed among IEGs such as *Egr1*, *Egr2*, and *ΔFosB* likely reflect coordinated transcriptional activation in response to stress. These genes are well-established markers of neuronal activity and synaptic plasticity, and their co-expression may indicate convergent regulation and roles in mediating stress-induced neural adaptations.

### 4.3 Study 1 - social defeat, CeA *Fkbp5* expression, and submission

Here, we also found increased CeA *Fkbp5* expression in acutely defeated rats (6 h post-defeat), and that greater *Fkbp5* expression is associated with briefer submission latencies in repeatedly defeated rats and greater CeA Fos induction after the final defeat. The latter result is consistent with the hypothesis that FKBP51 may modulate CeA responses to stress and complements human findings that *FKBP5* variants predict stress-induced amygdala activity (Holz et al., 2015; Pagliaccio et al., 2014, 2015a, 2015b). Interestingly, in previous work, deletion of *Fkbp5* in mice did not change anxiety-related, stress-coping, or depression-like behavior under basal conditions, but led to more active-coping behavior following acute stressors (Touma et al., 2011), including the forced swim test (Hartmann et al., 2012; Hoeijmakers et al., 2014). The collective results support the hypothesis that CeA FKBP51 is a key moderator of neural and behavioral responses to stress, here linked to social subordination.

In addition, in repeatedly defeated rats, *Fkbp5* expression positively correlated most strongly with both *Fos* and *Fosb* expression; all three genes also showed significant inverse correlations with latency to submit. These findings suggest that elevated *Fkbp5* expression associates with enhanced CeA neuronal activation, especially with induction of Fos family transcription factors, and faster social subordination during repeated social stress exposure. However, as our study was not designed to infer causality or directionality, these observations should be interpreted as correlative and for future causal, mechanistic study.

The correlation between *Fos* and *Fkbp5* expression observed in both unstressed controls and repeatedly defeated animals may suggest a coordinated regulation between neuronal activation and *Fkbp5* expression under both baseline and chronic stress conditions. In contrast, the lack of correlation following acute stress may indicate a transient stress-induced disruption of this association, which was restored after repeated defeat, potentially consistent with an adaptation, habituation, or other experience-dependent process. This speculation requires experimental testing.

### 4.4 Study 2 - relation of CeA Fkbp5 expression to “relapse” EtOH self-administration

FKBP51 has gained significant interest for its putative involvement in modulating alcohol drinking in humans (Dragan et al., 2018; Nylander et al., 2017) and rodents (Cruz et al., 2023; Dragan et al., 2018). Thus, we studied the CeA expression of *Fkbp5*, both canonical and truncated GR isoforms (*Nr3c1a, Nr3c1b*), H_1_ histaminergic receptors (*Hrhr1*), and IEG (*ΔFosB*) in a foot shock-based PTSD-relapse drinking model. In this behaviorally characterized cohort (Logrip and Zorrilla, 2012), mean CeA expression of *Hrhr1* gene was increased in foot shock-stressed rats relative to unstressed controls, while the other studied genes did not differ at the time of sacrifice, almost 2 months post-shock. Of note, antagonism of H_1_ receptors in the amygdala impaired experience-related fear behavior (Serafim et al., 2012), and pharmacological blockade and knockdown of H_1_ receptors in the bed nucleus of stria terminalis (BNST), which composes a major division of the extended amygdala along with the CeA, reduced anxiety-like behaviors in rats submitted to acute restraint stress (Li et al., 2023).

The lack of significant change in the expression levels of CeA *Fkbp5* and *Nr3c1* differs from our previous finding that these target genes remained elevated in the CeA, especially in males, 11 weeks following our recent 2-hit inhibitory PTSD/AUD model (Cruz et al., 2024). The apparently different outcome may reflect the more robust 3-mA foot shock, inhibitory avoidance conflict, or longer post-traumatic interval (*e.g.*, incubation) of the 2-hit model study (Cruz et al., 2024).

Even in the present study, inspection of Supplemental Fig. 4 did show a descriptive increase of ∼0.5 SD for *Fkbp5,* with a subset (*n* = 6) of stressed subjects showing levels >1 SD above those of controls. Additionally, high-reacquisition drinking animals in the present study expressed significantly higher CeA levels of *Fkbp5* than low-reacquisition drinking animals, and *Fkbp5* mRNA correlated directly with higher BECs. Interestingly, effect sizes for these relationships were similar in rats with or without a history of repeated foot shock, suggesting that the association to relapse-like self-administration does not require stress. GR, M_1_, M_3_ and H_1_ transcripts were not differentially expressed in relation to drinking level or BECs during reacquisition. However, we cannot rule out the possibility that benztropine's effects on the reacquisition of EtOH self-administration are also mediated through these targets, since altered receptor signaling may still occur without corresponding detectable changes in gene expression levels.

Interestingly, CeA *ΔFosB* expression was reduced in rats with pharmacologically relevant BECs compared to those with low BECs. One interpretation is that greater alcohol exposure may have an opposite effect on the expression of this gene compared to stress (as observed in the social defeat stress study). This may help explain the lack of differences between groups when comparing unstressed controls and foot shock-stressed animals without accounting for BECs.

High-relapse drinking has been conceptualized as a maladaptive, passive coping response to stress (Cooper et al., 1992). In humans, passive coping styles and negative life events interact to predict longitudinal alcohol use as, “… people scoring high on emotion coping, characterized by a passive, resigned, indulgent and self-accusatory coping style, increase their alcohol use after experiencing a negative life-event” (Veenstra et al., 2007). Several preclinical findings link passive coping responses, like subordination, to increased drinking. Subordinate rodents drink more EtOH than dominants in the visible burrow system (Blanchard et al., 1987; Wolffgramm and Heyne, 1991). In squirrel monkeys, dominance status inversely correlates with EtOH intake during social housing (McKenzie-Quirk and Miczek, 2008). Miczek et al., 2022. proposed that the social stress of subordination in the resident-intruder model can increase EtOH intake (Miczek et al., 2022). Conversely, rats with the “active coping” response to rapidly bury a shock probe showed reduced voluntary EtOH drinking (Sandbak and Murison, 1996). However, the relationship of coping style to drinking may be stressor-dependent or complex because in response to predator odor, only active, and not passive, behavioral responses predicted increased EtOH self-administration (Ornelas et al., 2021). Because greater CeA *Fkbp5* expression is associated with both faster defeat submission and increased reacquisition drinking, future studies can assess whether it is a shared molecular mechanism that links subordination or other passive coping responses to drinking.

Roozendaal, Koolhaas, and Bohus proposed over 30 years ago that the CeA subserves the selection of passive vs. active behavioral responses to stress because lesions of it reduced freezing, but not active burying, responses to a previously electrified shock-probe (Roozendaal et al., 1991). More recent work suggests that activation of somatostatin-positive neurons in the lateral CeA (CeL) promotes passive fear responses (*e.g.*, immobility, freezing), while competitive activation of mutually inhibitory somatostatin-negative CeL neurons (many that express protein kinase Cδ, enkephalin, and/or corticotropin-releasing factor [CRF]) instead reduces freezing and promotes active fear responses (*e.g.*, escape) (Fadok et al., 2017; Haubensak et al., 2010; Yan et al., 2019; Yu et al., 2016). Also, intra-CeA infusion of pituitary adenylate cyclase-activating polypeptide (PACAP), CRF, or vasopressin yielded shifts from active-to-passive coping styles under stress, whereas oxytocin receptor stimulation promoted active coping behaviors (Legradi et al., 2007; Pliota et al., 2020; Roozendaal et al., 1992). Single-cell RNAseq shows that CeA *Fkb5* is most highly and frequently expressed in non-somatostatin Supertypes (*e.g.*, 0368 high *Cartpt/Penk*, low *Sst*, 0370 high *Penk*, low *Sst*, 0373 high *Nts*, low *Sst*, 0375 high *Nts/Penk*, low *Sst*, and 0371 high *Cck/Penk*, low *Sst*). In comparison, lower *Fkbp5* expression is seen in high *Sst* Supertypes (*e.g.*, 0369, 0385, and 0386) (Atlas; van Velthoven et al., 2024). How this cell-type distribution relates to FKBP51's modulation of passive vs. active coping behaviors warrants study.

### 4.5 Study 3 - consideration of specificity of *Fkbp5* relations and benztropine effects

Expression of neither GR isoform (*Nr3c1a*, *Nr3c1b)* associated with reacquisition intake or BECs. These results show some specificity of CeA *Fkbp5* expression to reacquisition measures. Because it is well known that benztropine has non-FKBP51 actions and is classically considered a blocker of cholinergic and histaminergic signaling as well as dopamine reuptake (Rothman et al., 2008), we also studied *Chrm1, Chrm3,* and *Hrh1* to further explore specificity of CeA *Fkbp5* correlation to reacquisition EtOH measures. There was no significant correlation between the expression of these genes in the CeA and any drinking measure. In support of its FKBP51-modulating activity, benztropine increases GR activity in many human cell types, increases GR nuclear translocation in mouse neuronal culture via FKBP5 inhibition, and is selective for FKBP51 over its functionally opposing homolog, FKBP52 (Sabbagh et al., 2018; Sabban et al., 2018). Collectively, these findings support the notion that benztropine might reduce EtOH reacquisition drinking via FKBP51 inhibition, and not its other pharmacological targets. However, we cannot rule this out since we did not measure benztropine's targets in other relevant brain regions (*e.g.*, striatum). Future studies with more selective ligands can evaluate further the roles of FKBP51 vs. benztropine's other molecular targets in its anti-“relapse” effects.

### 4.6 Study 3 - comparison of benztropine actions on EtOH drinking to prior FKBP51 literature

In the foot-shock history relapse drinking model with both male and female rats, both benztropine doses (5 and 10 mg/kg) reduced “relapse” intake on the 2nd injection day, and the 10-mg/kg dose continued to reduce reacquisition drinking on the 3rd day. Similar to the present results with rat EtOH self-administration, SAFit2, a selective FKBP51 inhibitor, reduced relapse-like 2BC drinking in a male mouse model (Koenig et al., 2020). We (Cruz et al., 2023) and others (Koenig et al., 2020) previously have shown that FKBP51 inhibitors reduce alcohol intake using 2BC procedures in rodents. Conversely, other reports suggest opposite phenotypes in *Fkbp5* knockout mice, which exhibited increased EtOH consumption across several alcohol concentrations (Qiu et al., 2016b). Selectively bred high-drinking mice also did not show an effect of FKBP51 inhibition (Savarese et al., 2020). Perhaps the effects of current or past stress state influence the actions of FKBP51 inhibitors on drinking. We acknowledge that the absence of an unstressed control group is a limitation of our study. Future investigations should include unstressed groups to determine whether and how benztropine treatment interacts with stress exposure to influence the reacquisition of EtOH intake. The present results still show that benztropine can reduce EtOH reacquisition in rats with a history of stress exposure, which *per se* is of potential translational interest.

### 4.7 Study 3 - sex differences in benztropine action

Sex-specific analyses (considering males only or females only) suggested potential sex differences in benztropine action on EtOH relapse-like reacquisition drinking. In males, the 10 mg/kg dose reduced reacquisition intake on all days studied. In contrast, in female rats, benztropine only reduced EtOH self-administration vs. vehicle control on the 2nd and 3rd days of drug administration. In addition, both doses of benztropine (5 mg/kg and 10 mg/kg) decreased active EtOH responding in males compared to pre-extinction levels, while only the highest dose reduced this measure in females. While we speculate this reflects a greater response to benztropine in males than females, such conclusions must be made cautiously as the full model analysis did not show significant interactions involving Sex, perhaps due to low power to detect interaction effects. This pattern of results might relate to the higher basal vulnerability rates of drinking in females in this and other models, whereby females often display enhanced drinking compared to males (Steinman et al., 2021). Furthermore, previous research has shown that acute administration of a different FKBP51 antagonist, SAFit2, differentially reduced EtOH intake in stressed male, but not female, rats, similar to the present findings (Cruz et al., 2023). These and previous reports (Cruz et al., 2023; van Doeselaar et al., 2023) may suggest that some aspects of trauma and alcohol drinking are differentially mediated by FKBP51 mechanisms in males vs. females. Such differences may reflect that FKBP51 interacts not only with glucocorticoid receptors, but also with other steroid receptors, including progesterone, estrogen, and androgen receptors. FKBP51 has been suggested to inhibit progesterone receptor activity (Barent et al., 1998), while promoting estrogen and androgen activity (Shrestha et al., 2015; Stechschulte and Sanchez, 2011). Indeed, gonadal hormones may acutely moderate FKBP51 action because a rodent study demonstrated that an FKBP51 inhibitor only decreased stress-induced drug reinstatement at a timepoint when estrogen was low in females (metestrus/diestrus) (Connelly et al., 2020). Thus, future studies are needed to assess whether estrous stage or gonadal hormones influence benztropine actions on EtOH reacquisition. Future studies also are needed to understand *Fkbp5* expression in the CeA of stressed female rats, since only males were studied in the present defeat and post-shock gene expression analyses.

## 5 Conclusion

In summary, the present results are consistent with the hypothesis that CeA FKBP51 promotes passive stress-coping behaviors. Acute resident-intruder stress, which promoted subordination in the social defeat model, rapidly increased CeA IEG induction by 1 h and *Fkbp5* expression by 6 h post-defeat. Repeated defeat less robustly induced IEG activation than acute defeat, but the degree of continued CeA *Fos* expression occurred in strong, direct relation to *Fkbp5* expression. Both *Fkbp5* and *Fos* expression in the CeA correlated with faster defeat submission latency, and, in a model of EtOH relapse self-administration, greater CeA *Fkbp5* expression associated with greater alcohol intake and higher BECs. Benztropine mesylate (FDA-approved Cogentin®), which inhibits the association of FKBP51 to GR, dose-dependently reduced reacquisition of EtOH self-administration in rats with a stress history, and its effects were more robust in male vs. female rats. The collective results warrant causal study of CeA FKBP51 in passive stress-coping behaviors. They also call for the evaluation of selective FKBP51 inhibitors or, alternatively, antagonists of benztropine's other targets, to reduce alcohol relapse risk in individuals with a history of repeated, traumatic stress.

## Funding

Funding was provided through National Institutes of Health grants R01 AA028879, P60 AA006420, R21 AA029498, R01 AA027700, R21 MH124036, R37 AA017447, R01 AA029841, K99 AA030609, F32 AA018914, T32 AA007456, and K99 AA031718. The present study was also supported by the UCSD Triton Research & Experiential Learning Scholars and the Pearson Center for Alcoholism and Addiction Research.

## CRediT authorship contribution statement

**Luisa B. Bertotto:** Conceptualization, Data curation, Formal analysis, Investigation, Methodology, Visualization, Writing – original draft, Writing – review & editing. **Eleanna M. Sakoulas:** Conceptualization, Data curation, Formal analysis, Investigation, Methodology, Writing – original draft, Writing – review & editing. **Marian L. Logrip:** Conceptualization, Investigation, Methodology, Writing – review & editing. **Katrina Lin:** Investigation, Methodology, Writing – review & editing. **Anastasia E. Pimentel:** Investigation, Methodology, Writing – review & editing. **Lenwood Thompson:** Investigation, Methodology, Writing – review & editing. **Bryan Cruz:** Conceptualization, Writing – review & editing. **Valentina Vozella:** Conceptualization, Writing – review & editing. **Cristiane A. Favoretto:** Conceptualization, Formal analysis, Visualization, Writing – review & editing. **Marisa Roberto:** Conceptualization, Writing – review & editing. **Eric P. Zorrilla:** Conceptualization, Formal analysis, Funding acquisition, Methodology, Project administration, Resources, Supervision, Visualization, Writing – original draft, Writing – review & editing.

## Declaration of competing interest

The authors declare the following financial interests/personal relationships which may be considered as potential competing interests: Eric Zorrilla reports financial support was provided by 10.13039/100000002National Institutes of Health. Marisa Roberto reports financial support was provided by 10.13039/100000002National Institutes of Health. Luisa Bertotto reports financial support was provided by 10.13039/100000002National Institutes of Health. Bryan Cruz reports financial support was provided by 10.13039/100000002National Institutes of Health. Other authors declare that they have no known competing financial interests or personal relationships that could have appeared to influence the work reported in this paper.

## Data Availability

Data will be made available on request.
